# Substantial Nitrogen Oxide Pollution Is Embodied in the Bilateral Trade between China and the European Union

**DOI:** 10.3390/ijerph18020675

**Published:** 2021-01-14

**Authors:** Yan Li, Yigang Wei, Xueqing Wang, Hanxiao Xu

**Affiliations:** 1 Business School, Shandong University, Weihai 264209, China; liyan5@sdu.edu.cn; 2 School of Economics and Management, Beihang University, Beijing 100191, China; 3 Beijing Key Laboratory of Emergency Support Simulation Technologies for City Operations, Beijing 100191, China; 4 School of Management and Economics, Tianjin University, Tianjin 300072, China; 201700620080@mail.sdu.edu.cn; 5 School of Environment and Natural Resources, Renmin University of China, Beijing 100872, China; 201700810285@mail.sdu.edu.cn

**Keywords:** production and consumption ends, embodied NO_x_ emissions, China–EU trade, MRIO model, NO_x_ emission reduction

## Abstract

Against the backdrop of globalization and trade facilitation, the products consumed by a country are more and more relying on the importation of those products from other countries. Therefore, the pollutant emissions of products associated are transferred from consuming countries to exporting countries, which significantly changes the spatial distribution of global pollutant emissions. The objective of this research is to analyse the embodied nitrogen oxide (NO_x_) emissions in the trading process between China and the European Union (EU) and to further trace the interindustry and intercountry transfer paths. This study constructs a multiregional input–output (MRIO) model based on the latest EORA global supply chain database. The MRIO model quantitatively analyses the total NO_x_ emissions from the production and consumption ends of China and the EU from 1995 to 2014. Important findings are derived from the empirical results as follows. (1) In 2014, China’s production end emissions were 1824.38 kilotons higher than those of the consumption end. By contrast, the situation in the EU was the opposite, i.e., production end emissions were 1711.97 kilotons lower than those of the consumption end. (2) In the trade between China and the EU, the EU is a net importer of embodied NO_x_, and China is a net exporter of embodied NO_x_. In 2014, 2.55% of China’s domestic NO_x_ emissions were transferred to the EU in China-EU trade, accounting for 2.75% of China’s domestic consumption demand. (3) In 2014, Electricity, Gas and Water (397.75 kilotons), Transport (343.55 kilotons), Petroleum, Chemical and non-metallic Products (95.9 kilotons), Metal Products (49.88 kilotons), Textiles and Apparel (26.19 kilotons), are among the industries with the most embodied NOx emissions from China’s net exports during its two-way trade with the EU. (4) In the bilateral trade between the EU and China, many countries are in the state of embodied NO_x_ net import. The top three net importers in 2014 were Germany (169.24 kilotons), Britain (128.11 kilotons), France (103.21 kilotons).

## 1. Introduction

The relationship between trade and environment has become a research hotspot with more and more attention paid to global climate change. Trade activities themselves will increase the scale of production and the consumption of resources, resulting in more pollution emissions and pollution country transfer [1]. Global trade in NOx pollution is high and the transfer of embodied NOx between major economies is large. Embodied NOx trade has become a vital statistical indicator in international environmental studies. The nitrogen footprint and its effects on the environment and health have elicited growing research attention in recent years [2,3,4,5]. The destructive effects of nitrogen oxide (NO_x_) emissions on the environment are largely embodied in the following aspects. In addition to causing primary pollution, NO_x_ exhibits high chemical activity, produces photochemical smog and damages the ozone layer, further intensifying the greenhouse effect [6]. Furthermore, once NO_x_ is dissolved in water, nitric acid rain is produced, possibly causing further widespread environmental damage, huge economic losses, corroding buildings and industrial equipment and the yield reduction or even death of crops [7]. Regardless of the considerable reduction in sulphur dioxide emissions due to the efficient control of air pollution in China, the decline in NO_x_ emissions is still nonideal, and NO_x_ remains as one of the most harmful and challenging atmospheric pollutants to deal with. With the rapid development of the economy, controlling air pollution caused by NO_x_ is an urgent issue [8].

**Figure 1:**
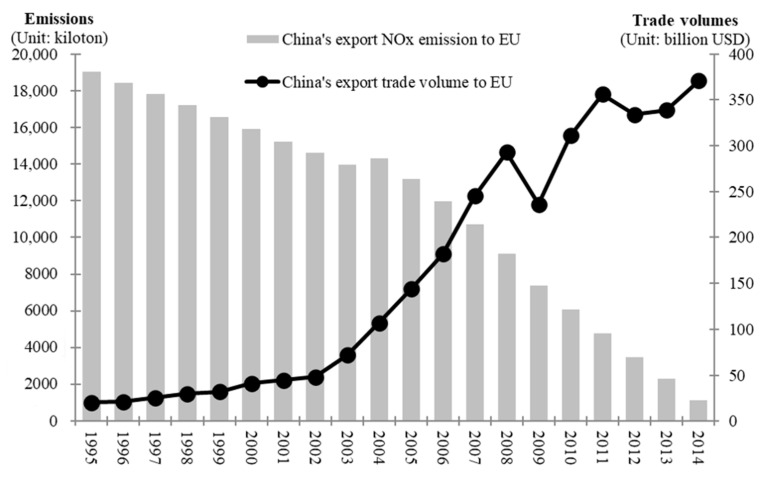
Export volume from China to the EU and the embodied NO_x_ emissions (Left Axis Unit: kiloton; Right Axis Unit: billion USD. Data Sources: UNCTAD Database).

China’s foreign trade is an important driving force for the development of the national economy [9]. Since China joined the WTO in 2001, its foreign trade has entered a new stage. The volume of imports and exports of commodities exceeded US $1 trillion in 2004, US $2 trillion in 2007 and US $3 trillion in 2011, and US $4 trillion in 2013 (China’s Ministry of Commerce, 2019). In the same year, China surpassed the US to become the world’s largest trade country. Europe plays an important role in global foreign trade. From 2004 to 2014, the EU surpassed Japan and the US and was China’s largest trading partner for ten consecutive years, while China was the EU’s second largest trading partner. In 2014, China and the EU promoted the comprehensive strategic partnership and trade grew rapidly. China’s export volume to the EU increased from 20.376 billion US dollars in 1995 to 370.8 billion US dollars in 2014. EU’s export volume to the China increased to 244.3 billion US dollars in 2014 (China’s Ministry of Commerce, 2015). China–EU trade is highly significant to the development of both economies and the global economy. However, the rapid growth of trade between China and the EU and the expansion of China’s trade surplus have stimulated the emission of air pollutants. As the largest energy consumer, China emits a considerable amount of air pollutants, such as CO_2_, NO_x_, sulphur dioxide (SO_2_) and fine particulate matter (PM_2_._5_). Figure 1 indicates that with the rapid growth of China’s export volume to the EU, the emission of implied NOx from China’s export to the EU showed a downward trend year by year. From 2001 to 2008, the average annual growth rate of China’s export volume to the EU was 30.9%, which dropped in 2009, and the average annual decrease rate of the implied NOx exports in the same period was 8.2%. From 2009 to 2011, China’s export volume to the EU continued to increase, with an average annual growth rate of 22.7%. In 2012, it declined slightly, followed by a slow growth trend. In the same period, the export volume of NOx decreased at an average annual rate of 19.7%. The air pollution caused by the embodied NO_x_ emissions exerts a negative impact on the ecosystem [10] and poses a considerable threat to human health [11,12]. Air pollution in China has elicited worldwide attention, and public calls to control air pollution are increasing [13]. The development of China’s foreign trade, coupled with its long-term status as a world processing region, has led to the export of many manufactured goods from China to other countries, including many energy- and pollution-intensive products, resulting in massive fossil energy consumption and increasing the emission of air pollutants. This cross-border movement of air pollutants is becoming increasingly serious [14]. Under these circumstances, issues related to air pollutants embodied in China’s foreign trade have elicited further attention. Foreign trade leads to the cross-border movement of air pollutants. The EU is the largest trading partner of China, and NO_x_ is one of the most harmful and challenging atmospheric pollutants to deal with. Therefore, the embodied NO_x_ in China–EU trade should be analysed.

With the rapid growth of economic globalisation, the impact of international trade on the environment has attracted considerable attention from all countries. Shan et al. [15] used social media data to analyse the emotional responses of people to river pollution from the aspects of trends, seasons, space and dynamics (TSSD). Environmental pollution has become an urgent problem, and China bears high levels of environmental pollution for other countries in international trade. In recent years, an increasing number of studies have found that developed countries typically transfer their carbon emissions [16], biodiversity threats [17], ozone precursors [18] and land utilization [19] to developing countries through foreign trade. Huang et al. [20] determined that the United States (US), the EU and Japan transfer their domestic pollution to China through bilateral trade, making China a ‘pollution paradise’ for developed countries. Wu et al. [21] estimated the CO_2_ emissions of China and Japan from 2000 to 2009. The result showed that China is the net exporter of the embodied CO_2_ in the China–Japan trade, and the export trade is the major driver of the increase in embodied CO_2_ emissions. Zhong et al. [22] studied sulphur oxides transferred through foreign trade and found that sulphur oxides mostly flow from developing countries, such as China, to highly developed economies, such as the US, the EU and Japan. Ju et al. [23] reported that in the trading process with the three largest economies of Asia (China, Japan and South Korea), China exports a considerable amount of embodied PM_2_._5_ but imports less embodied PM_2_._5_ emissions from other countries. A similar conclusion was drawn from most of the studies on issues of China’s embodied pollutant transfer; that is, China is indirectly responsible for a large amount of pollution emissions from other countries, particularly from developed countries, through international trade.

The principal objectives of current studies on pollutants embodied in foreign trade are air pollutants, such as CO_2_, SO_2_ and PM_2_._5_. Amongst which, CO_2_ was first used by domestic and foreign scholars to study pollution emission transfer embodied in foreign trade [24]. Li et al. [25] used cluster analysis to decompose China’s CO_2_ emission reduction tasks. Li et al. [26] forecast China’s CO_2_ emission intensity towards 2030.Besides, there are also some studies on the impact of emissions on other objects like energy consumption [27] and natural gas [28]. On the basis of the input–output model, Yang et al. [29] calculated the embodied carbon emissions in the trade between China and South Korea from 2000 to 2011. They concluded that trade between China and South Korea is beneficial for reducing pressure on China to decrease carbon emissions. Liang et al. [30] quantified the impact of consumption in 189 countries on global economic output and human health associated with PM_2_._5_. Their result showed that developed countries transfer PM_2_._5_ to Asian countries, such as China and India, through foreign trade. Zhong et al. [22] calculated the global production and consumption lists of sulphur oxides and studied the impact of foreign trade on the distribution of pollutant emission reduction obligations. Through the combination of a multiregional input–output database with the data of the Swedish economic and environmental accounts, Palm et al. [31] analysed the utilisation of land, water and material resources and the emissions of greenhouse gases, SO_2_ and PM_2_._5_. They concluded that apart from the use of land and material resources, Sweden is a net importer of all environmental pressures. Therefore, existing studies have focused on pollutants, such as CO_2_, PM_2_._5_ and sulphur oxides, and studies on NO_x_ should be enriched.

The input–output model is the primary method used to study the embodied pollution transfer in trade. This is a consensus that the input–output analysis method can be used to measure the embodied pollution in trade. Compared with the single-region input–output (SRIO) model, the MRIO model can internalise the import relationship of each country’s production industry on the basis of their input–output sheets, forming the input–output relationship involving multiple industries in multiple countries (or regions) and accurately estimating the impact of trade on global or regional emissions. At present, this method is widely used in various environment fields. For example, Veiga et al. [32] studied the embodied pollution in Brazilian trade at the country level by using the extended MRIO model. Janis et al. [33] analysed the equivalent CO_2_ emissions of the three countries in the Baltic states that were related to consumption in 1995–2011 on the basis of the MRIO model. At the industrial level, Suo et al. [34] analysed the embodied pollution emissions of trade between provinces (municipalities directly under the central government) and industries in the Beijing–Tianjin–Hebei region and surrounding areas in 2012 on the basis of the MRIO model. Applying the MRIO model, Pang et al. [35] established an MRIO sheet comprising 13 industries and four regions (Beijing, Tianjin and Hebei Provinces), showing that the industrial distribution pattern in the Beijing–Tianjin–Hebei region is the major factor that affects trans-provincial pollutant transfer. At the provincial level, Sun et al. [36] calculated the grey water footprint of the intermediate and end consumptions of 17 industries in China’s 30 provinces from 2002 to 2012 on the basis of the MRIO model. Zhu et al. [37] calculated carbon emissions in China’s provinces and regions to solve the climate trade dilemma via decarbonisation through industrial technology improvement based on the MRIO model. Their results indicated that with the improvement of critical data quality, such as input–output and bilateral trade, the MRIO model, which exhibits high accuracy, is widely used in the literature, providing an important basis for guiding the reduction of global pollution emissions.

Although the previous research results provide valuable references for the research perspective, contents and methods, the following deficiencies remain. (1) From the research perspective, previous studies have only calculated the transfer amount of trade pollution from each country and region from the perspective of total volume. By contrast, this study calculated the embodied NO_x_ emissions of the production and consumption ends from the total volume perspective and then further traced the embodied NO_x_ industry source from specific industry and country perspectives. (2) From the perspective of research content, previous studies have mostly focused on carbon emissions, water consumption patterns and the division of responsibilities amongst countries by trade, but insufficient attention was given to pollutants, such as NO_x_. By contrast, most of the studies on embodied pollutants calculate the total amount, flow direction and industrial distribution of embodied pollutants in major trades from a unilateral perspective, making the comprehensive description of embodied pollution transfer in bilateral trade difficult.

The objective of this study is to systematically analyse the embodied NO_x_ emission in the trading process between China and the EU from 1995 to 2014 combined with environment accounts and the input–output table in the EORA global supply chain database and then further trace the resources of industries where NO_x_ originates from the perspectives of industries and countries. In the current study, the MRIO model between China and the EU is established in accordance with the following concept: the total quantity of pollutants embodied in China and the EU—the transfer of pollutants embodied in the bilateral trade between China and the EU. NOx, the representative conventional air pollutant, is used as the research object. The following specific issues are investigated: (1) from the perspective of total volume, the dynamic change in embodied NO_x_ emissions in China and the EU based on the production and consumption ends; (2) from the perspective of industry chain, the transfer of the embodied industry in the bilateral trade between China and the EU and (3) from the country perspective, the transfer path of the embodied NO_x_ in China and the major member countries of the EU.

The contributions of this study are reflected in the following aspects. Firstly, in terms of research perspective, existing studies are mostly limited to the analysis between a country and trade unions as a whole. By contrast, this study analyses the embodied NO_x_ emissions between China and major member countries of the EU. Secondly, in terms of research content, NO_x_ is one of the most harmful and most challenging atmospheric pollutants to deal with [38]. Given the rapid economic development, effective control of air pollution caused by NO_x_ is urgent. At present, most studies on embodied pollutants calculate the total quantity, flow and industry distribution of embodied pollutants in major trades from the unilateral perspective. In the current study, the total quantity of NO_x_ embodied in the China–EU bilateral trade is set as the research objective to provide evidence for the reduction of NO_x_ emissions and the formulation of relevant environmental policies. This study provides experience for adjusting the industrial structure of developing countries in foreign trade to deepen the understanding of NO_x_ emission reduction.

## 2. Materials and Methods

### 2.1. Data

#### 2.1.1. Data Source

Data analysis was conducted on the basis of the input–output table of trade emissions between China and the EU; the data were obtained from EPRA global supply chain database. This database contains the world input–output table (WIOT) data of 189 countries (regions) and the rest of the world. The data span is 1995–2014. In addition, emissions from various industries in 28 member countries of the EU and China were obtained from the environment accounts in EORA. Meanwhile, the industrial characteristics of NO_x_ emissions were analysed in accordance with WIOT.

#### 2.1.2. Data Processing

The Eora global supply chain database contains 26 industries. Table 1 lists the 26 industries.

### 2.2. Research Method

#### 2.2.1. Construction of the MRIO Model

The MRIO table reflects the internal correlation between the inputs and outputs of each product (or department) at a certain time. The multiregional environmental input–output model is the most commonly used environmental accounting tool from the perspective of consumers. It is also the primary tool for measuring the environmental impact of international trade.

In a noncompetitive input–output model, the input–output relationship of a country is:

(1) $$\sum_{j\;=\;1}^{n}a_{\mathrm{ij}}X_{j}{+\;y}_{i}{\;=\;X}_{i},\;i\;=\;1,\;2,\;3\;\ldots \;n$$

where $a_{\mathrm{ij}}$ is the direct input of the I department required by a unit of output for the consumption of the j department, The calculation method of $a_{\mathrm{ij}}$ is as follows: removing the value of goods or services directly consumed by the product sector (or industry) in the production and operation of the product sector (or industry) by using the total input ${\;X}_{j}$ of product sector j or industry sector. $y_{i}$ is the ultimate demand of the i department, ${\;X}_{i}$ is the gross output of the i-th department. The MRIO model can be extended on the basis of the noncompetitive input–output table of one country. Suppose N countries (or regions) are included. The MRIO model can be represented as:

(2) $$\left(\begin{array}{c} X^{A}\\ \cdots \\ X^{N} \end{array}\right)\;=\;\left(\begin{array}{ccc} A^{\mathrm{AA}} & \cdots & A^{\mathrm{AN}}\\ \vdots & \ddots & \vdots \\ A^{\mathrm{NA}} & \cdots & A^{\mathrm{NN}} \end{array}\right)\left(\begin{array}{c} X^{A}\\ \cdots \\ X^{N} \end{array}\right)\;+\;\left(\begin{array}{c} Y^{A}\\ \cdots \\ Y^{N} \end{array}\right)$$

where the column vectors on the left of the equal sign are each department of country Q (departments 1, 2..., *n*), for a total output of *n*. The first term on the right of the equal sign is the A matrix, i.e., the direct consumption matrix that reflects the final demand for national products consumed per unit of output in each sector of country Q.

The combination of Equations (1) and (2) showed that the MRIO model reflects the input–output relationships amongst various sectors of the country and amongst various sectors of each country. The matrix form of Equation (2) is:

(3) X=AX+Y

(4) (I – A)X = Y

where the Leontief inverse matrix is L = ${\left(I\;–\;A\right)}^{-1}$, and I is the unit matrix. Then, Equation (3) can be represented as:

(5) X = LY

#### 2.2.2. Pollution Emission Accounting of Production and Consumption Ends

Since the differences in the production efficiencies between China and the EU, production of the same quantity of a certain product may result in different amount of embodied NOx. To take this fact into consideration, this article assumes that the emission factors of the two regions are the same.

We use $E^{Q}\;$ to represent the direct pollution emission coefficient of country Q, i.e., the pollution emission amount generated per unit of output. Then, the MRIO model can be used to describe pollution transfer within regions. $E^{Q}$ is the column vector composed of the direct emission coefficient considering the different production technology levels of each department:

(6) $$E^{Q}{\;=\;(e}_{1}^{Q}{,\;e}_{2}^{Q},\;\cdots {\;e}_{n}^{Q})$$

The global pollution direct emission coefficient vector E denotes the direct emission coefficients of *N* countries composed of the column vector. Referring to the complete consumption coefficient, the global pollution complete emission coefficient can be further constructed as $E_{p}$, which can be expressed by the following equation:

(7) $$E_{p}{\;=\;E(I\;-A)}^{-1}\;=\;\mathrm{EL}$$

where the complete emission coefficient of country Q is $E_{p}^{Q}$, and the ultimate output matrix of country Q is $Y^{Q}$. Then, $E_{p}^{Q}$ of the $E_{p}$ diagonalised matrix $\hat{E_{p}^{Q}}$ is further introduced, and $\hat{E_{p}}$ will obtain the pollution emission matrix of country Q on the basis of production accounting:

(8) $${\mathrm{PO}}^{Q}\;=\hat{{\;E}_{p}^{Q}}Y^{Q}$$

The ultimate demand column vector of region Q is $D^{Q}$. Then, the pollution emission matrix of region Q is obtained on the basis of consumption accounting ${\mathrm{DO}}^{Q}$:

(9) $${\mathrm{DO}}^{Q\;}=\;\hat{E_{p}}D^{Q}$$

where the corresponding elements in ${\mathrm{DO}}^{Q}$ and ${\mathrm{PO}}^{Q}$ of region Q is ${\mathrm{PO}}_{i}^{\mathrm{QS}}\;$ and ${\;\mathrm{DO}}_{i}^{\mathrm{SQ}}$, which respectively represent the trading pollution caused by the commodities transferred from industry I in region Q to region S and the embodied pollution caused by the commodities transferred from region S to the i-th industry in region Q. The pollution emissions of the production and consumption ends of region Q can be obtained on the basis of ${\mathrm{PO}}^{Q}$ and ${\;\mathrm{DO}}^{Q}$.

## 3. Research Results and Discussion

### 3.1. Embodied Emissions of NO_x_ in China and the EU Based on the Accounting of the Production and Consumption Ends

The pollution emissions of the production and consumption ends are respectively based on ‘producer responsibility’ and ‘consumer responsibility’ to divide the responsibility of the pollution emissions of all parties. Under the production end accounting system, a country will be solely responsible for emissions caused by its own production, regardless of whether the product is consumed domestically or exported. Under the consumption end accounting system, all pollution emissions caused by a country’s ultimate demand are attributed to that country, including domestic and foreign emissions caused by ultimate demand [8]. Both production and consumption end emissions actually reflect the ‘production capacity’ and ‘consumption capacity’ of a country for a pollutant. When the entire world is considered, production end emissions are equal to consumption end emissions. However, when a specific country is considered, the production and consumption capacities of pollutants are no longer the same, and the transfer of embodied pollutants is inevitable due to the different manners, statuses and depths of each country’s participation in international trade, and the differences in the technological level and energy efficiency of each country [39]. The difference between pollution emissions from the production and consumption ends is the net export embodied pollution. A positive difference indicates that the region is the net export area of the embodied pollution. A negative difference indicates that the region is the net import area of the embodied pollution.

**Figure 2:**
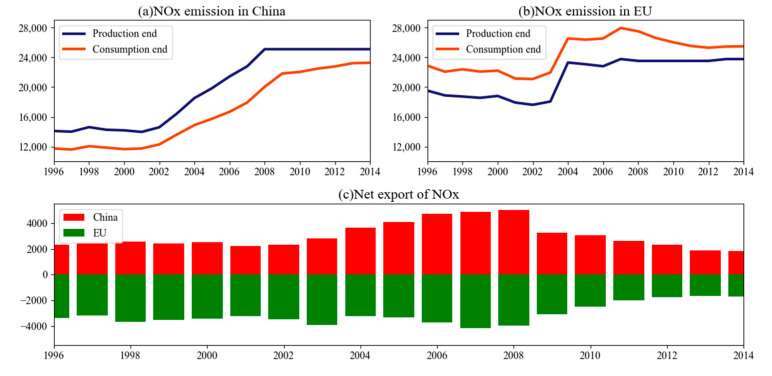
NO_x_ emission amounts of China and the EU based on the production and consumption ends (Unit: kiloton).

From the preceding method, the embodied NO_x_ emissions of China and the EU were calculated on the basis of production and consumption end accounting, and the results for the period of 1995–2014 is presented in Figure 2, suggesting that China’s production end emissions during this period exhibited a rapid growth, increasing from 13,847.01 to 25,091.34 kilotons, i.e., an increase of 81.2%. In particular, after China’s accession to the World Trade Organization (WTO) in 2001, a large increase in production end emissions occurred, with emissions increasing by 11,117.25 kilotons from 2001 to 2014, accounting for approximately 98.87% of the total growth during the entire research period. China’s NO_x_ emissions based on the production end are significantly higher than those based on the consumption end. In 2014, China’s production end emissions were 1824.38 kilotons higher than those of the consumption end. By contrast, the situation in the EU was the opposite, i.e., production end emissions were 1711.97 kilotons lower than those of the consumption end. Therefore, the EU is a net importer of embodied NO_x_, and China is a net exporter of embodied NO_x_. In general, a certain difference will arise in the accounting results of the NO_x_ emissions of a country based on the production and consumption ends, particularly for typical export-oriented economies, such as China and the EU. In 2008, China’s NO_x_ emissions (25,091.34 kilotons) exceeded that of the EU (23,519.68 kilotons) based on production end accounting. By contrast, the emissions of the EU (27,497.18 kilotons) were higher than those of China (20,046.28 kilotons) based on consumption end accounting. Thus, the accounting method based on the production end disregards the NO_x_ emission transfer embodied in export goods. As a major importer of embodied NO_x_ during trades, the EU avoids considerable NO_x_ emissions through international trade. By contrast, as a major exporter of embodied NO_x_, China indirectly assumes a large amount of emission responsibility for other countries under this accounting system.

Figure 2a,b show the NOx emissions in China and the EU from the production end and the consumption end respectively, and Figure 2c shows the change tendency of the embodied NO_x_ in the net exports of China and the EU from 1995 to 2014. The net export embodied NO_x_ of each country and region was calculated on the basis of the difference between the production and consumption end emissions of the country or region. For China, the embodied NO_x_ of its net exports during the study period exhibited a steady–rise–fall trend. In 1995–2002, the net export embodied NO_x_ of China was relatively steady, but it rapidly increased since 2002, reaching the highest value of 5045.06 kilotons in 2008, with an annual increase rate of 13.2%. However, the 25,091.34 kilotons of production end NO_x_ emissions of China in 2008 indicated that the 20.11% NO_x_ emissions produced by domestic production activities was consumed by other countries as a result of net exports. From 2009 to 2014, The China’s net export of embodied NOx has been consistently decreased. The rise of net export embodied NO_x_ represents a continuing imbalance between China’s NO_x_ production and consumption, which is highly related to the expansion of trade surplus, the increase in foreign investment and the transfer of backward production capacity from developed countries to China. Meanwhile, the decline in net export embodied NO_x_ in 2009–2014 was partly due to the decline in net exports caused by the global economic crisis and partly due to the rise in NO_x_ consumption caused by China’s stimulus of domestic demand. With regard to the EU, which is always the net importer during the study period, its foreign emissions caused by its own consumption exceeded the domestic emissions borne by exports. The EU’s net import of embodied NO_x_ has been consistently above 3000 kilotons (in 1995–2007, The net import of NOx in the EU showed a relatively stable trend from 1995 to 2007, and showed a sharp decline from 2007 to 2014, with an average annual growth rate of −12%. During the whole study period, the net import of NOx in the EU peaked at 4174.08 kilotons in 2007, By contrast, the production end NO_x_ emission of the EU was 23,763.84 kilotons that year, indicating that 17.56% of the NO_x_ emissions produced by the domestic demand of EU countries were paid by other countries.

**Figure 3:**
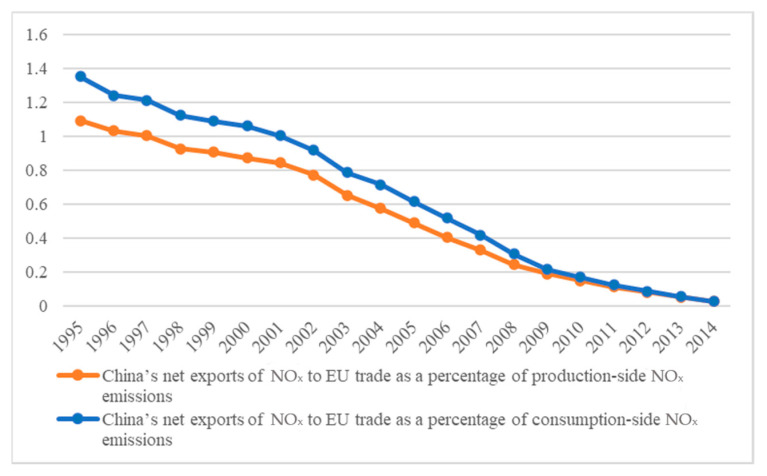
Percentage of China’s net exports of NOx in trade with the EU.

Furthermore, Figure 3 shows the proportion of China’s net exports of NOx in the trade with the EU on its own production-side NOx emissions and consumption-side NOx emissions. During the entire study period, China’s net exports of NOx to the EU as a percentage of China’s production and consumption emissions have shown a downward trend year by year. From 1995 to 2002, the rate of decline was slow, and the average annual growth rate was −4.82% and −5.40% on the production side and consumption side, respectively. The rate of decline began to accelerate in 2003, and the average annual growth rates from 2003 to 2014 were −25.52% and −26.28%. In 2014, China’s net exports of NOx to the EU were 643.88 kilotons. In the same year, China’s production-side emissions were 25,091.34 kilotons, indicating that 2.55% of China’s total NOx production was transferred to the EU when considering China’s trade with the EU. This part accounted for 2.75% of China’s own consumption demand. It can be seen that the EU, as one of China’s larger trading partners, enjoys net exports of implied pollutants from China. An average of 53.79% of China’s domestic NOx emissions are transferred to the EU in China-EU trade.

Overall, China is a net exporter of embodied NO_x_ and the EU is a net importer. The EU avoids NO_x_ emissions through international trade. By contrast, China indirectly bears much of the responsibility for other countries’ emissions.

### 3.2. Analysis of Embodied NO_x_ Industrial Transfer Path in the Bilateral Trade between China and the EU

#### 3.2.1. Aggregate Analysis of Embodied NO_x_ Emissions in the Bilateral Trade between China and the EU

China’s foreign trade has developed rapidly since joining the World Trade Organization. Therefore, this paper selects 2001, 2008, and 2014 as the nodes to conduct a general analysis of the total amount of implied NOx emissions in trade between China and the EU. As an important export region, the embodied NO_x_ emissions in China’s export to the EU in 2001, 2008 and 2014 (15,242, 9091 and 1141 kilotons) accounted for 5.2%, 5.3% and 4.7% of China’s domestic emissions and for 20.1%, 22.1% and 21.1% of the total embodied NO_x_ emissions of China’s exports (75,825 thousand, 41,557 thousand and 5414 kilotons), respectively. The world MRIO tables for 2001, 2008 and 2014 show that China’s exports to the EU increased by more than USD 300 billion between 2001 and 2014. However, contrary to the rapid growth of China’s export trade volume to the EU, the implied NOx emissions of China’s exports to the EU decreased by 14,101 kilotons during the same period, and the proportion of total domestic emissions also decreased from 5.2% to 4.7%. In the bilateral trade between China and the EU, China bore more responsibilities for NO_x_ emissions than the EU for the production of commodity exports. The figures reached 55,655 kilotons in 2001, 12,600 kilotons in 2008 and 1563 kilotons in 2014. This gap reflected the following facts: (1) the EU transfers their own pollution to China by importing a large number of processed commodities from China and (2) the large amount of EU’s foreign direct investment in China, which is concentrated in ‘high pollution, high emission’ areas. The EU has realised large-scale NO_x_ transfer through its bilateral trade with China, making it one of the major beneficiaries of the embodied NO_x_ of China’s export.

#### 3.2.2. Industrial Transfer Path in the Bilateral Trade between China and the EU

**Figure 4:**
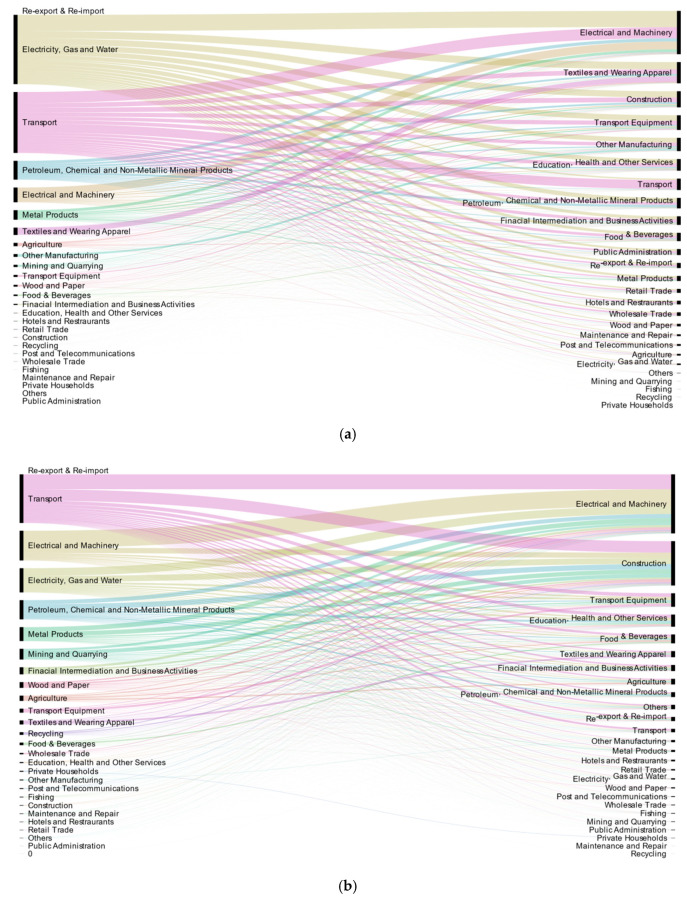
Industry transfer of the embodied NO_x_ emissions in the bilateral trade between China and the EU in 2014. (**a**) Industry transfer of NO_x_ emitted from China to the EU; (**b**) Industry transfer of NO_x_ emitted from the EU to China. (The sender on the left and the receiver on the right).

Figure 4a with the export perspective shows that the embodied NO_x_ of China’s export to the EU in 2014 was mostly aggregated in the industries of electricity, gas and water (401.25 kilotons, accounting for 36.16%); transport (352.82 kilotons, accounting for 30.92%); petroleum, chemical and non-metallic mineral products (1108.93 kilotons, accounting for 9.55%); electrical and machinery (85.47 kilotons, accounting for 7.49%) and metal products (55.32 kilotons, accounting for 4.85%). Figure 4b shows that exports from the EU to China were mostly concentrated on transport (129.65 kilotons, accounting for 26.1%), electrical and machinery (79.57 kilotons, accounting for 16%), electricity, gas and water (64.9 kilotons, accounting for 13.05%), petroleum, chemical and non-metallic mineral products (51.42 kilotons, accounting for 10.34%), and metal products (37.36 kilotons, accounting for 7.5%). The industrial structure indicates that some energy-intensive industries, such as electricity, gas and water belonging to secondary industries (401.25 kilotons, 36.16% of the total), contribute the most to the embodied NO_x_ emissions.

From the import perspective, the imported embodied NO_x_ (497.28 kilotons) in various industries of China in 2014 was generally small compared with that of the exports (2282.31 kilotons). From the industry distribution shown in Figure 4b, the electrical and machinery (157.59 kilotons, accounting for more than 31.69%), construction (118.75 kilotons, accounting for more than 23.88%), transport equipment (36.22 kilotons, accounting for more than 7.28%), education, health and other services (32.58 kilotons, accounting for more than 6.55%) and food & beverages (23.85 kilotons, accounting for more than 4.8%) industries of the EU import the largest amount of NO_x_ to China. Figure 4a shows that the top five industries of the EU that import the most NO_x_ from China are the electrical and machinery (250.83 kilotons, accounting for more than 22%), textiles and wearing apparel (122.94 kilotons, accounting for more than 10.77%), construction (93.54 kilotons, accounting for more than 8.2%), transport equipment (85.14 kilotons, accounting for more than 7.5%) and other manufacturing (76.84 kilotons, accounting for more than 6%) industries. By contrast, 33.68% of the NO_x_ imported by the textile industry in the EU comes from China’s electricity, gas and water industries. Because the development of the textile industry depends on the supply of electricity, the EU’s textile industry is more dependent on China’s electricity, gas and water.

In general, 10 industries were in the state of embodied NOx net export in the bilateral trade between China and the EU in 2014. In the process of bilateral trade with the EU, industries with high NOx emission embodied by net exports are electricity, gas and water (397.75 kilotons), transport (343.55 kilotons), petroleum, chemical and non-metallic mineral products (95.9 kilotons), metal products (49.88 kilotons), textiles and apparel (26.19 kilotons).

### 3.3. Analysis of the Embodied NO_x_ Transfer in China and the Member Countries of the EU

**Figure 5:**
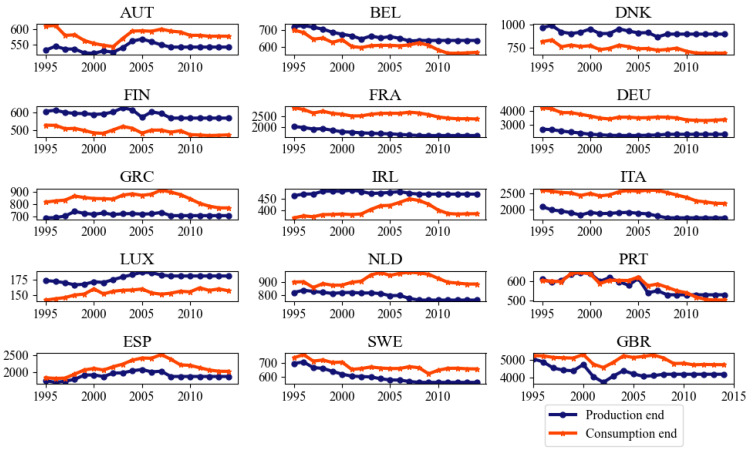
Production and consumption end emissions of 15 EU member countries during the period of 2001–2014 (Unit: kiloton).

Figure 5 shows that from the production and consumption ends of EU member countries from 2001 to 2014, the UK’s production end NO_X_ emissions ranked first in the EU, reaching 4193.78 kilotons in 2014. The top five EU production end emission countries are the United Kingdom, Germany, Italy, Spain, and France. In 2014, the production end emissions reached 4193.78, 2280.68, 1876.59, 1736.76 and 1601.96 kilotons. The top five emitters in the consumption end in the EU are the UK, Germany, France, Italy and Spain, whose consumption end emissions in 2014 reached 4720.84, 3377.15, 2386.19, 2188.66 and 2028.56 kilotons, respectively. Ireland and Luxembourg are at the bottom of the list in the production and consumption ends.

A detailed analysis was performed on 15 EU member countries on the change tendency of their production and consumption ends from 2001 to 2014. The NO_x_ emissions from the production end of 12 countries, namely, Belgium, Germany, Finland, Spain, the UK, France, Ireland, Italy, the Netherlands, Portugal, Luxembourg, Greece and Sweden, were generally stable or decreasing. Austria’s production end emissions were on the rise from 2002 to 2005, reaching a peak of 567.88 kilotons in 2005, with an average annual growth rate of 2.7%, and on the decline from 2006 (558.32 kilotons) to 2008 (540.32 kilotons). Denmark’s production end emissions exhibited a steady upward and downward trend, rising rapidly from 2002, reaching a peak of 951.8 kilotons in 2003, with an average annual growth rate of 5.4%. Since then it began to decline and reached its lowest point in 2007 (867.6 kilotons). The analysis of the consumption end indicated that the UK grew steadily with an average annual growth rate of 1.47%, from 2002 to 2007. Meanwhile, Italy and Spain grew steadily with an average annual growth rate of 1.2% and 3.4%, respectively, from 2001 to 2007. And changes in other countries during this period are relatively stable. Except for Sweden and Finland, the consumption end of the remaining EU’s 13 member countries demonstrated an average annual growth rate of NO_x_ emissions from 2009 to 2014. Among them, the average annual growth rates of Britain, Germany, France, Italy, and Spain from 2007 to 2014 were −1.5%, −0.8%, −1.8%, −2.4%, and −3.1%.

**Figure 6:**
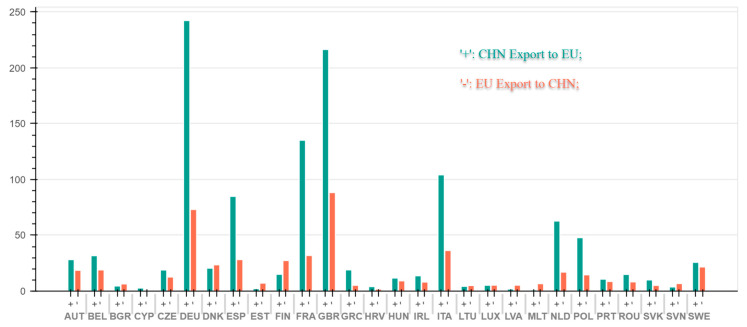
Embodied NOx flows in bilateral trade between China and 28 EU countries in 2014. (The x-axis represents EU countries, and the y-axis represents NO_X_ emissions) (Unit: kiloton).

As can be seen from Figure 6, the top six countries in China’s export of embodied NOx emissions to 27 EU countries in 2014 were Germany (242.31 kilotons), the United Kingdom (216.34 kilotons), and France (135.07 kilotons), Italy (104.07 kilotons), Spain (84.78 kilotons), the Netherlands (62.7 kilotons).The top six countries in the EU’s NOx emissions from 27 EU countries are the United Kingdom (88.23 kilotons), Germany (73.07 kilotons), and Italy (36.33 kilotons), France (31.86 kilotons), the Spain (28.15 kilotons), Finland (27.33 kilotons).In the bilateral trade between the EU and China, Bulgaria, Denmark, Estonia, Finland, Latvia, Lithuania, Luxembourg, Malta, Slovenia are in the state of embodied NOx net export, the rest of all is in the state of embodied NOx net import. The top six net importers in 2014 were Germany (169.24 kilotons), the UK (128.11 kilotons), France (103.21 kilotons), Italy (67.73 kilotons), Spain (56.62 kilotons) and the Netherlands (45.74 kilotons). The top two net importers, namely, Germany and the UK, are selected here for a detailed analysis.

At present, China has been Germany’s largest trading partner for the third consecutive year since 2018, and Germany has become China’s largest trading partner in Europe. Germany is currently China’s largest source of technology in Europe based on data released by the German federal statistics office. From the perspective of industry transfer, the top five industries with the most embodied NO_x_ emissions from China’s exports to Germany were electricity, gas and water (85.51 kilotons, accounting for 35.29%); transport (73.86 kilotons, accounting for 30.48%); petroleum, chemical and non-metallic mineral products (23.11 kilotons, accounting for 9.5%); electrical and machinery (16.56 kilotons, accounting for 6.8%) and metal products (12.11 kilotons, accounting for 5%) industries. At present, China’s imports of NO_x_ emissions from Germany are mostly concentrated in value-added technologies and capital-intensive industries. In 2014, the imported embodied NO_x_ of electrical and machinery accounted for 33.82% of the total import embodied NO_x_. In the same year, the proportion of embodied NO_x_ in the total volume of embodied NO_x_ in exports was 16.56%, and the proportion of embodied NO_x_ in imports was significantly higher than that in exports because the electrical and machinery industry of Germany is relatively developed, and a large number of German electrical and machinery equipment was imported by China to meet its requirements of economic development. The embodied NO_x_ emitted by the import of low value-added products accounted for a lower proportion of total imports. For example, the low value-added textile and leather footwear industry’s import embodied NO_x_ accounted for only 1.46% of the total import embodied NO_x_ emissions in 2014. In the same year, the proportion of the embodied NO_x_ in the total emissions of embodied NO_x_ in exports reached 10.28%, which was caused by China’s large textile exports to Germany and its relatively backward textile technology.

The UK officially left the EU in 2019, but remained a member of the EU during the study’s sample period. As shown in Figure 5, the analysis of the industrial transfer indicated that the top five industries with embodied NO_x_ emissions in China’s imports from the UK are the electrical and machinery (28.28 kilotons, accounting for 32.05%); construction (21.35 kilotons, accounting for 24.2%); transport equipment (6.4 kilotons, accounting for 7.25%); education, health and other services (6.36 kilotons, accounting for 7.2%) and food & beverages (3.86 kilotons, accounting for 4.4%) industries. The top five industries with embodied NO_x_ emissions in China’s exports to the UK are transport (73.35 kilotons, accounting for 34%); electricity, gas and water (72.58 kilotons, accounting for 33.55%); petroleum, chemical and non-metallic mineral products (19.19 kilotons, accounting for 8.87%); metal products (9.36 kilotons, accounting for 4.3%) and textiles and wearing apparel (7.26 kilotons, accounting for 3.3%). These industries are the same as two of the top five industries with NO_x_ emissions embodied in China’s imports to the UK. Departments with a high percentage of NO_x_ emissions embodied in the summary of import and export trades are closely related to China’s processing trade. At present, China’s processing trade with the UK firstly imports raw materials and components from the UK for domestic assembly and processing and then exports processed industrial semi-finished or finished products to the UK. This trade mode produces a large amount of embodied NO_x_ in the import and export trades of these industries.

In general, China’s exports to the EU are energy-intensive products, such as the electricity, gas and water; transport and petroleum, chemical and non-metallic mineral products industries. China’s imports from the EU are primarily capital- and technology-intensive products. For high value-added products, China, as a developing country, has a relatively low standing in producing technology and achieving energy efficiency, resulting in a large amount of embodied NO_x_ net exports.

## 4. Conclusions and Policy Recommendations

A considerable embodied emission transfer occurs against the booming trade between China and the EU. Accordingly, an MRIO model was constructed in this study to calculate the embodied emissions in the trade between China and the EU, which analyses the embodied NO_x_ transfer from the perspectives of the production and consumption ends, industry and country to formulate a reference for emission reduction, trade and environmental protection policies.

The major conclusions are as follows:

(1) From an temporal perspective, the emissions of China’s production and consumption ends from 1995 to 2014 increased to a certain extent in the bilateral trade between China and the EU, China’s production end emissions during this period exhibited a rapid growth, increasing from 13,847.01 to 25,091.34 kilotons, i.e., an increase of 81.2%. Moreover, production end emissions have been consistently higher than those of the consumption end. After China’s accession to the WTO, the gap between the two rapidly widened, reaching 1824.38 kilotons in 2014. By contrast, the situation in the EU was the opposite, i.e., production end emissions were 1711.97 kilotons lower than those of the consumption end.
(2) From an overall trade perspective, in Sino-European trade, China is a typical net exporter of embodied NO_x_, and the EU is a net importer of embodied NO_x_. From China’s perspective, in 2014, China’s net exports of NOx during trade with the EU accounted for 2.55% and 2.75% of its own production-side NOx emissions and consumption-side NOx emissions. Between 1995 and 2014, an average of 53.79% of China’s domestic NOx emissions were transferred to the EU in China-EU trade.
(3) From an industry perspective, ten industries were in the state of embodied NOx net export in the bilateral trade between China and the EU in 2014. In the process of bilateral trade with the EU, industries with high NOx emission embodied by net exports are electricity, gas and water (397.75 kilotons), transport (343.55 kilotons), petroleum, chemical and non-metallic mineral products (95.9 kilotons), metal products (49.88 kilotons), textiles and apparel (26.19 kilotons).
(4) From a country perspective, in the bilateral trade between the EU and China, many countries are in the state of embodied NOx net import. The top six net importers in 2014 were Germany (169.24 kilotons), Britain (128.11 kilotons), France (103.21 kilotons), Italy (67.73 kilotons), Spain (56.62 kilotons) and The Netherlands (45.74 kilotons).

The aforementioned conclusions provide rich policy implications. (1) Emission control over heavily polluting industries should be strengthened and China’s ability and technology to reduce pollution should be enhanced. Given that trade in industries such as transportation and auxiliary equipment manufacturing and electricity, natural gas and water supply will deteriorate China’s ecological environment, focus on improving its pollution control ability and on strengthening the remediation of motor vehicles are necessary. (2) At present, China’s industries with high energy consumption and emissions account for a relatively high proportion. Therefore, effort should be exerted to change its industrial structure, phase out backward production capacity, improve the green level of industries and gradually transfer from heavy chemical industries that produce high pollution and emissions to strategic emerging industries with high added value and low emissions.

## Figures and Tables

**Table 1:** Industries after consolidation.

Serial Number	Industry	Serial Number	Industry
1	Agriculture	14	Construction
2	Fishing	15	Maintenance and Repair
3	Mining and Quarrying	16	Wholesale Trade
4	Food & Beverages	17	Retail Trade
5	Textiles and Wearing Apparel	18	Hotels and Restraurants
6	Wood and Paper	19	Transport
7	Petroleum, Chemical and Non-Metallic Mineral Products	20	Post and Telecommunications
8	Metal Products	21	Finacial Intermediation and Business Activities
9	Electrical and Machinery	22	Public Administration
10	Transport Equipment	23	Education, Health and Other Services
11	Other Manufacturing	24	Private Households
12	Recycling	25	Others
13	Electricity, Gas and Water	26	Re-export & Re-import

## Data Availability

Data sharing is not applicable to this article.
